# Barriers, Benefits and Complications of Orthodontic Treatment in Patients with Epidermolysis Bullosa: A Patient-Reported Cross-Sectional Study

**DOI:** 10.3390/healthcare14111584

**Published:** 2026-06-04

**Authors:** Sebastián Véliz, Gudrun Salamon, Milica Kabic, Sophie Strobl, Pedro Diz-Dios, Colomba Besa-Witto, Susanne Krämer

**Affiliations:** 1 Department of Periodontology, Dental Clinic, Sigmund Freud University, 1020 Vienna, Austria; sebastian.veliz@med.sfu.ac.at; 2 Facultad de Medicina y Odontoloxia, Universidad de Santiago de Compostela, 15782 Santiago, Spain; 3 Special Care Dentistry Unit, Faculty of Dentistry, University of Chile, Santiago 8380544, Chile; 4 Health Lab, Centre for Medical and Health Psychology, Faculty of Psychology, Sigmund Freud University Vienna, 1020 Vienna, Austria; 5 Department of Dermatology, Medical Centre, Medical Faculty, University of Freiburg, 79098 Freiburg im Breisgau, Germany

**Keywords:** epidermolysis bullosa, orthodontic treatment, barriers, complications, benefits

## Abstract

**Introduction**: Epidermolysis Bullosa (EB) is a rare genetic condition with skin and mucosal fragility. Patients with EB present extra- and intraoral manifestations that can limit their access to dental treatment, including orthodontic treatment. This research aims to determine the barriers, benefits and complications of orthodontic treatment reported by a group of patients living with EB. **Materials and Methods**: This observational study included *n* = 101 patients with a genetic diagnosis of EB. After their regular dental consultation, they were interviewed about factors they considered barriers to accessing orthodontic treatment. Those who underwent orthodontic treatment (*n* = 24) reflected about their perceived benefits and complications from the therapy. Data were analysed with descriptive statistics and multiple Fisher’s exact tests with false discovery rate (FDR) correction. **Results**: The most prevalent barrier was that most patients with EB had never been evaluated by an orthodontist (74.3%), even if the teams had an orthodontist. Other barriers included distance to the treatment centre (42.6%), poor oral hygiene (27.7%) and poor oral health status (26.7%). Patients with limited mouth opening perceived greater treatment benefit compared to those without this limitation (φ = −0.28, *p* = 0.0242), while an increasing age was associated with a reduced perception of treatment benefit (Cramér’s V = 0.29, *p* = 0.0404). Among those who underwent orthodontic treatment, the most prevalent benefits of orthodontic treatment were aesthetic improvement (62.5%), oral hygiene improvement (20.8%) and occlusal stability (12.5%), while the most prevalent complications were wounds and ulcers (75.0%), gingivitis (54.1%), poor oral hygiene (41.6%) and caries (33.3%). **Discussion**: People living with EB reported different barriers to orthodontic treatment relating to psychosocial and professional aspects, which vary according to the EB type and severity. The involvement of orthodontists in multidisciplinary special care teams and the reduction in access barriers to dental specialities can be facilitated by a more comprehensive understanding of conditions such as EB.

## 1. Introduction

Epidermolysis Bullosa (EB) is a rare genetic disorder with skin and mucosa fragility caused by variants in at least 16 genes that encode structural proteins of the dermo-epidermal junction [1]. The present classification considers four major types, namely EB Simplex (EBS), Junctional EB (JEB), Dystrophic EB (DEB) and Kindler EB (KEB). The complexity of this classification stems from the possibility that distinct phenotypes might be caused by the same gene, and similar phenotypes may be attributed to different genes. For this reason, EB presents a highly variable clinical phenotype with more than 30 subtypes, ranging from mild to severe cases [1,2].

The primary clinical manifestation of EB is the appearance of lesions in the skin and the mucosa (such as ulcers, blisters and wounds) [1,2]. Patients with mild subtypes may present minor skin anomalies, such as nail dystrophy; while those with severe subtypes, such as Severe Recessive Dystrophic EB (RDEB), can present with chronic pain, pseudosyndactyly and acral deformities, high incidence of squamous cell carcinoma (SCC), gastrointestinal and nutritional anomalies, ocular abnormalities, anaemia, cardiomyopathy and renal impairments among several other complications [1,2,3,4].

Oral findings in EB also vary according to the type and subtype [5]. While patients with EBS and Dominant DEB (DDEB) present with occasional oral lesions, people living with RDEB presents with more frequent oral lesions, strictures (including microstomia, ankyloglossia and vestibule obliteration), loss of tongue papillae and palatal rugae and poor oral hygiene associated with a high risk of caries [5,6,7,8]. Patients with JEB can present with perioral granulation tissue, syndromic amelogenesis imperfecta (SAI), crown resorption and teeth retention [5,9,10,11]. Kindler EB is mainly associated with early onset periodontal disease and some types of SAI [5,12,13,14].

Malocclusions are more prevalent in RDEB. Shah et al. reported that patients with RDEB present with reduced facial growth and suggested that this impairment is associated with malnutrition and scarring, contributing to dentoalveolar disproportion and, consequently, dental crowding [15]. Several case reports have shown successful approaches to orthodontic treatment, including teeth extractions, fixed metallic braces, aligners and mini implants [16,17,18,19,20,21,22,23], leading to the development of clinical practice guidelines for orthodontic treatment in people with EB [24]. These reports mention several complications and barriers faced by patients with EB in relation to accessing and receiving orthodontic treatment, including treatment adaptation requirements [21], needing a multidisciplinary approach [17,19,22,23] and accessing treatment [20].

There are no published patient-reported studies exploring the barriers, benefits and complications of orthodontic treatment in EB. The existing evidence is limited to case reports and one Clinical Practice Guideline (CPG) based on experts’ opinions, none of which used patient-reported outcomes [16,17,18,19,20,21,22,23,24]. It has been well reported that patients with disabilities, despite showing a high prevalence of malocclusion and orthodontic treatment needs, face barriers to accessing orthodontic treatment. These can be related to the patients (e.g., difficulties during orthodontic care), their caregivers (e.g., caregivers’ burden) or medical professionals (e.g., lack of clinical instruction treating special care patients) [25,26,27,28]. This study aims to determine the barriers patients living with EB perceive to receiving orthodontic treatment and the benefits and complications during the treatment.

## 2. Materials and Methods

An observational study was conducted at the Special Care Dentistry clinic, Faculty of Dentistry, University of Chile, in association with DebRA Chile. The study included all patients with a genetic diagnosis of EB registered at DebRA Chile who received dental treatment at the Faculty of Dentistry of the University of Chile between January 2023 and December 2024. All patients from the national register were potential participants, as dental treatment is granted for all patients with EB due to an agreement with the University of Chile. The invitation to participate was made through DebRA Chile. Informed consent was obtained from all participants. Patients with EB who either did not want to participate or did not follow instructions were excluded. This project was approved by the Ethics Committee of the North Metropolitan North Health Service, Chile (002/2023). It did not have additional funding. This report follows Strengthening the Reporting of Observational Studies in Epidemiology (STROBE) guidelines for cross-sectional studies [29].

Data collection was conducted in Spanish in two steps: for the first step, as part of their regular dental consultation, an orthodontist evaluated all parameters, assessing oral health status, presence of malocclusions and need for orthodontic treatment. For the second step, one of the researchers [S.V.] conducted a semi-structured interview with the patients about their experience regarding orthodontic treatment and the barriers they may have faced (Supplementary File S1). The responses were coded by the interviewer into predefined categories. No open-ended qualitative analysis was performed. Those patients who had received orthodontic treatment were additionally asked about their perceived benefits and complications.

The barriers reported by patients were part of an exploratory statistical analysis. First, potential associations between the EB patients’ barriers and sociodemographic characteristics (gender, age, and EB type) were explored. With regard to the four main EB types, DEB was split into RDEB and DDEB and handled as separate groups due to their markedly different oral phenotypes [5]. Next, an analysis was conducted to determine whether there were any structural correlations between the individual barriers. A further exploratory analysis was conducted to determine the extent to which patients’ self-assessment of their oral hygiene corresponded with the assessment of the examining professional.

Potential sources of bias were identified. Selection bias may be considered from the exclusion of participants that declined participation or could not attend during the examination period, potentially leading to underrepresentation of those individuals with more severe disease or less engagement with healthcare services. Recall bias and socially desirable bias can be considered in a self-reported design. To address these possible sources, a semi-structured interview was used. The questionnaire categories were extracted from the EB-Ortho, part of the EB-Oral Health Assessment Form, a content-validated instrument used at the centre. Additional biases are discussed in Section 4.3. No formal sample size calculation was performed due to the design of the study, including all participants from the national reference centre. According to the number of participants included (*n* = 101), a confidence level of 95% and an expected proportion of 50%, this sample size allows an estimate of approximately 6.7% margin of error.

Due to the small sample size and unbalanced distributions of several dichotomous variables, all correlation analyses were performed using Fisher’s exact test. The Phi coefficient (φ) was reported as the effect size measure for 2 × 2 contingency tables and Cramér’s V for larger tables. Since all barriers examined were dichotomously coded, the Phi coefficient also corresponds to the Pearson correlation for 2 × 2 contingency tables and can thus be consistently interpreted as a correlation and effect size measure in the context of the Fisher test with two dichotomous variables. The analyses were exploratory in nature; therefore, conservative control of the α error (e.g., Bonferroni correction) was not performed. Instead, we applied the false discovery rate (FDR), as defined by Benjamini and Hochberg [30]. Applying a correction for multiple testing irrespective of the underlying research question may lead to overly conservative results. In other words, while the probability of a false-positive finding is reduced, the probability of a false-negative finding may increase. Therefore, the tests were conducted in two separate “test families”, i.e., two corrections were performed for two different research questions: (A) the correlations between barriers and sociodemographic characteristics, and (B) the pairwise correlations between the barriers themselves.

## 3. Results

### 3.1. Sample Description

**Figure 1:**
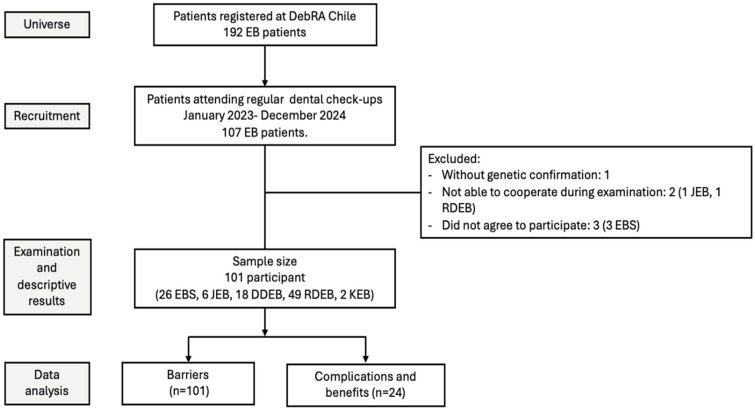
Flow diagram of participants. EBS: Epidermolysis Bullosa Simplex; JEB: Junctional Epidermolysis Bullosa, DDEB: Dominant Dystrophic Epidermolysis Bullosa, RDEB: Recessive Dystrophic Epidermolysis Bullosa, KEB: Kindler Epidermolysis Bullosa.

The convenience sample comprised *n* = 101 patients with EB, all attending dental treatment between January 2023 and December 2024 (Figure 1). This sample corresponds to 52.6% of all patients registered at the Chilean national registry [31]. The sample included 54 male (53.5%) and 47 female patients (46.5%), with a mean age of 22.2 years (SD ± 15.4; range 2–74). The distribution by type and subtype of EB includes 26 patients with EBS from three subtypes: localised (*n* = 3), intermediate (*n* = 18) and with muscular dystrophy (*n* = 5); six patients with JEB from two subtypes: intermediate (*n* = 3) and severe (*n* = 3); 18 patients with DDEB (Dominant DEB) from two subtypes: localised (*n* = 14), and pruriginous (*n* = 4); 49 patients with RDEB from four subtypes: localised (*n* = 1), intermediate (*n* = 21), severe (*n* = 25) and inversa (*n* = 2); and two patients with KEB (Table 1). A total of six participants were excluded or declined to participate, according to Figure 1 (94.4% response rate).

### 3.2. Self-Reported Barriers to Orthodontic Treatment in People Living with EB

The self-reported barriers were classified into four groups, as seen in Table 1 and in the extended version (Supplementary Table S1): EB-related and oral health barriers, psychosocial barriers, professional barriers, and others. Overall, the most prevalent barriers related to EB and oral health were poor oral hygiene (*n* = 28, 27.7%), poor oral health status (*n* = 27, 26.7%) and limited mouth opening (*n* = 18, 17.8%). These three characteristics were mainly prevalent in patients with severe RDEB. Concerning the psychosocial barriers, the most prevalent barrier was “difficulties accessing the clinic” (*n* = 43, 42.6%), highly prevalent in patients with EBS with muscular dystrophy (*n* = 5, 100%) and patients with severe RDEB (*n* = 20, 80.0%). The barrier “did not consider it necessary or did not see the benefit” (*n* = 27, 26.7%) was highly mentioned in patients with EBS and DDEB, with 42.3% and 44.4%, respectively. Additionally, 74.3% of the patients (*n* = 75) noted that this was the first time an orthodontist evaluated them. Six patients mentioned additional barriers that were grouped together under “others”, such as treatment length, reluctance of the caregiver, lack of EB-trained professional and drug abuse.

EB types showed significant differences across several barriers (see Table 2). Patients with RDEB reported limited mouth opening, poor perceived oral hygiene, and generally poor oral health status more frequently than patients with other EB types. Compared to other EB types, they also reported significantly more problems related to difficulties accessing the clinic. At the same time, RDEB patients perceived the treatment as beneficial. In contrast, sociodemographic analyses revealed no significant effects of gender or age on perceived barriers. However, increasing age was associated with a reduced perception of treatment benefit (Cramér’s V = 0.29, *p* = 0.0404).

**Figure 2:**
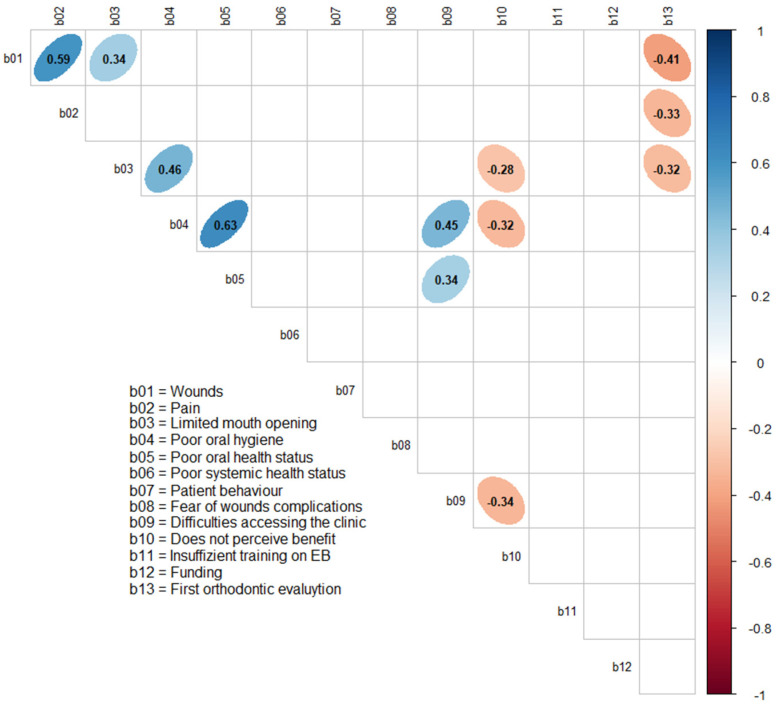
Significant bivariate correlations between barriers.

Figure 2 illustrates the significant associations between barriers. Overall, a higher number of wounds was associated with greater pain (φ = 0.59, *p* < 0.01) and with limited mouth opening (φ = 0.34, *p* = 0.0274). Patients reporting more wounds (φ = −0.41, *p* = 0.0039) and pain (φ = −0.33, *p* = 0.0242) were also more likely to have undergone orthodontic treatment in the past. A significant association was observed between limited mouth opening and poor oral hygiene (φ = 0.46, *p* < 0.01). Furthermore, patients with limited mouth opening perceived greater treatment benefit compared to those without this limitation (φ = −0.28, *p* = 0.0242). Poor oral hygiene was associated with poorer overall oral health status (φ = 0.63, *p* < 0.01) and with difficulties accessing the clinic (φ = 0.45, *p* < 0.01). Patients with poor overall oral health status did not perceive treatment as beneficial (φ = 0.34, *p* = 0.0117); however, this pattern was not observed among patients reporting poor oral hygiene alone. Finally, difficulties accessing the clinic were significantly associated with those who perceived orthodontic treatment as beneficial (φ = −0.34, *p* < 0.01).

Notably, significant discrepancies were observed between perceived and clinically assessed oral health status (φ = 0.41, *p* < 0.001). Only 27% (*n* = 28) perceived a poor oral health status, while clinical examination revealed that 94% (*n* = 94) had some degree of periodontal disease. Therefore, the results must be interpreted with caution.

### 3.3. Benefits of Orthodontic Treatment in People Living with EB

Of the sample (*n* = 101), 24 patients had received orthodontic treatment prior to or during the time of the assessment. The most frequently reported benefit of orthodontic treatment was aesthetic improvement (*n* = 15, 62.5%), followed by oral hygiene improvement (*n* = 5, 20.8%) and occlusal stability (*n* = 3, 12.5%). Other reported benefits included fewer ulcers after orthodontic treatment (*n* = 2, 8.3%), improved oral function, more space for the tongue, and orthodontic traction of a fractured tooth that allowed proper oral rehabilitation, with each of these reported by one patient (*n* = 1, 4.1%) (Table 3).

### 3.4. Complications During Orthodontic Treatment in People Living with EB

In terms of oral health, the most prevalent complications were wounds or lesions (*n* = 18, 75.0%), gingivitis (*n* = 13, 54.1%), poor oral hygiene (*n* = 10, 41.6%) and caries (*n* = 8, 33.3%). In terms of complications of orthodontic treatment, the most prevalent was debonding of brackets or appliances (*n* = 7, 29.1%), archwire complications (*n* = 5, 20.8%), suspension of the treatment (*n* = 5, 20.8%) and non-compliance with appliance instructions (*n* = 4, 16.6%). One patient with RDEB had poor compliance with follow-up appointments due to EB-related health issues. From this subsample, only four patients (16.6%) mentioned having no complications during the treatment. The distribution by type and subtype is shown in Table 3.

## 4. Discussion

The present study highlights the self-reported perceived barriers that people living with EB can face regarding orthodontic treatment, as well as its associated benefits and complications.

### 4.1. Barriers to Orthodontic Treatment for People with EB

The World Health Organization defines a barrier as “a factor in a person’s environment that, through their absence or presence, limits functioning and creates disability” [32]. In this study, several aspects are considered as limiting the access to timely orthodontic treatment by people living with EB.

The most prevalent barrier was that despite their age and type of EB, patients had never been evaluated by an orthodontist, even though an orthodontist was a regular team member. This can be considered a local factor, due to the national reference centre having an orthodontist in their team, something that not many EB reference centres have. Additionally, not all patients visit the centre regularly, especially those with moderate to mild forms of EB. This can be partially explained by the second most prevalent barrier: difficulties accessing the clinic. Many patients must travel to receive dental treatment with professionals specialised in EB; therefore, economic burden, time constraints and location can play a crucial role in attending appointments [5], mostly in groups with mobility impairment such as severe RDEB and EBS with muscular dystrophy. This can be considered a significant barrier to orthodontic treatment, as appointments tend to be scheduled on monthly basis and the treatment may last months or even years. This may lead patients to opt for teeth extractions over orthodontic treatment [20].

It had been previously stated that patients with RDEB can present significantly higher scores for plaque, gingival inflammation and caries (Decay–Missing–Filled Teeth index; DMFT), leading to complications such as pain or infections that consequently lead to teeth extractions, making orthodontic treatment a lower priority or impossible to provide [5]. Poor oral hygiene when combined with orthodontic appliances and tooth movement forces can significantly compromise periodontal health [33]. Interestingly, a significant difference was found between clinically assessed and self-perceived oral health. Although many patients presented with poor oral health indices, few considered this to be a barrier for orthodontic treatment. Moreover, many participants who considered orthodontic treatment not necessary, presented a clear need for orthodontic treatment. This suggests that, since orthodontic treatment does not seem necessary to them, aspects such as poor oral health is not considered a barrier. Studies have shown that the concordance between self-reported and clinically assessed oral health indices varies considerably in the general population, with ranges between 52% and 65.4% [34]. Studies exploring the relationship between self-perceived and clinically assessed oral health status are needed in the EB population.

Participants reporting more wounds and pain were more likely to undergo orthodontic treatment, but contrary to what one may think, wounds and pain presented a low prevalence (*n* = 8, 7.9%) as a barrier, even below mouth opening limitation (*n* = 18, 17.8%), which was only reported by patients with RDEB. An association was found between mouth opening limitation and poor oral hygiene observed in participants with RDEB, in line with previous reports [5].

Overall, the results suggest that perceived barriers are related to the severity of EB manifestations. Patients with RDEB were the most severely affected, particularly due to limited mouth opening and compromised oral hygiene and oral health. Despite these substantial challenges, they perceived the treatment as beneficial and had received orthodontic treatment significantly more often, even though accessing the clinic was associated with considerable difficulties. The structural relationships among the different barriers further support this interpretation and can largely be explained by EB type, reflecting overall disease severity.

The significant association between older age and reduced benefit perception is consistent with studies exploring motivations for orthodontic treatment among healthy adults, which showed that, for some of them, orthodontic treatment is not a priority but rather a secondary motivation after achieving other life milestones [35]. Still, these factors should be assessed within a model considering contextual factors, such as personal and environmental elements. Internationally, costs have repeatedly been reported as a central barrier. For example, people living with orofacial clefts (OFCs) in the United States face several economic barriers to accessing proper orthodontic treatment [36]. In the present sample, patients have access to low-cost orthodontic treatment through an agreement between DebRA Chile Foundation and University of Chile, turning “financial barrier” into a low-prevalence barrier (*n* = 7, 6.9%). Nevertheless, financial coverage remains donation-based and is not anchored in the public health system.

Expertise in special care dentistry is crucial for ensuring a good care situation. Patients with rare diseases or special care needs often find it difficult to access orthodontic treatment due to a poor understanding of their condition among medical professionals. In patients with neurodevelopmental disorders, barriers identified during orthodontic treatment include the caregivers’ fear that the patient would not be able to complete the orthodontic treatment because of their behaviour; meanwhile, the residents reported needing more chair time to treat this group and that their clinical instructors were not prepared to treat patients with neurodevelopmental disorders, which may compromise the attitude of the residents towards treating patients with disabilities or different conditions in the future [25,37]. In “other barriers”, one participant reported as a barrier that the professional was not properly trained for treating people living with EB. This was mentioned by one patient who used to receive orthodontic treatment and moved to another country, where the specialists could not continue the treatment and removed the brackets; therefore, centres without a trained orthodontist may have a higher prevalence in this barrier. However, this barrier also seems to work in reverse: it has been reported that orthodontists and orthodontic residents have low levels of confidence and preparation when it comes to treating patients with special needs, craniofacial malformations and developmental delays, and that those with previous experience are more willing to treat these patients [25,37]. Regarding EB, the European Reference Network for Rare Diseases (ERN) included in their 2024 Oral Health Care Pathways for patients with EB that several specialists, including an orthodontist, must evaluate patients at a high risk for oral and dental manifestations and complications during childhood [38]. This is intended to secure timely management of malocclusions, as patients with RDEB have an increased prevalence of malocclusion due to reduced craniofacial growth and high prevalence of crowding, and those with JEB have to be monitored for dental anomalies such as retained teeth [5,15,38].

### 4.2. Benefits and Complications of Orthodontic Treatment for People with EB

Orthodontic treatments benefit special care patients in several aspects, including better appearance and function, including chewing, speaking and drooling control; all these factors lead to better self-esteem and social acceptance [28,39,40,41]. In our study, aesthetic improvement was the most frequently reported benefit, followed by oral hygiene improvement and occlusal stability. Furthermore, improvement in oral function and more space for the tongue were also identified. Very specific for EB, a decrease in the number of ulcers after improving tooth alignment was mentioned as a benefit by two patients with a history of traumatic ulcers caused by malocclusion. This is very relevant for improving oral pain in patients with EB.

Oral ulcers are a key feature of EB, related to mucosal and skin fragility. This fragility and the concern of orthodontic appliances causing even more damage has always been risen by patients, parents and health professionals when orthodontic treatment is mentioned. The evidence of orthodontic treatment causing ulcers in healthy population is frequently reported as a complication, with percentages ranging from 63.6% [42] and 50.5% [43] to 0% [27]. In a study of patients with rare diseases undergoing orthodontic treatment, ulcers as an oral complication were described in 7.4% [27]. In our sample, unsurprisingly, wounds or lesions were the most common complication reported by 18 patients (75%), despite the suggestion of using orthodontic wax to manage it [22]. To date, no article has established a number of ulcers caused by orthodontic treatment in people with EB in comparison to how many would occur even without treatment.

Patients in our cohort also presented higher prevalence of gingivitis (*n* = 13, 54.1%), poor oral hygiene (*n* = 10; 41.6%) and caries (*n* = 8, 33.3%) when compared to patients with other rare diseases under orthodontic treatment, who reported 31.9% of patients with gingivitis and 6.4% of patients with caries [27]. This can be explained by the increased precision required for oral hygiene when using orthodontic appliances such as brackets [44], which is extremely challenging in our patients with severe forms of EB such as RDEB, due to pseudosyndactyly, microstomia, vestibule obliteration and ankyloglossia [5]. Proper oral hygiene has been reported to be difficult to maintain in special care patients under orthodontic treatment [28] and is even more complex in EB. Therefore, patients with EB under orthodontic treatment require more preventive measures, including adjuvants and fluoride varnish. During the orthodontic treatment, the frequency of follow-up appointments with preventive measures should increase when compared to those periods without brackets [5].

The most frequent technical complication regarding orthodontic treatment was spontaneous debonding of the appliances or brackets, affecting 29.1% of participants (*n* = 7). Similar prevalence has been reported in patients with rare diseases (30.9%) compared to systemically healthy individuals (12.8%) [27]. In our study, this can be related to factors such as amelogenesis imperfecta in patients with JEB and microstomia in patients with RDEB, as these characteristics hinder the effectiveness of bonding agents and limit proper appliances’ impression, design and bonding [21,23]. Treatment suspension was necessary for five patients, four with RDEB and one with DDEB, for various reasons: one patient could not tolerate the pain caused by orthodontic tooth movement, one presented several traumatic lesions associated with discomfort and pain, one presented systemic health complications due to renal failure, one suspended the treatment after moving abroad to a country where he could not find a professional that could treat him, and the patient with DDEB lost a removable appliance several times. Orthodontic treatment in patients with rare diseases may require temporary or definitive treatment suspension for various reasons [27]; in our case, only one of five suspensions was attributed to the EB response to orthodontic treatment. Additionally, only one patient (4.1%) presented a systemic complication due to EB (renal failure), which led to treatment suspension. Even though this is a low-prevalence complication, it has been previously reported in patients with rare diseases with a similar frequency (3.2%) [27].

Overall, the benefits of orthodontic treatment in people living with EB tend to be lasting or permanent in nature, such as aesthetic improvement, oral hygiene improvement, and occlusal stability. These benefits can improve different physical and psychosocial aspects, including oral function and self-esteem. In contrast, most complications reported can be considered transitory, as these can resolve upon adjustment or removal of the appliances, such as wounds, gingivitis and poor oral hygiene. Permanent structural damage may occasionally occur due to caries or enamel demineralisation, highlighting the importance of involving patients in intense preventive programmes during the orthodontic treatment. The therapeutic value of orthodontic treatment in people living with EB, including risk/benefit assessment, should be viewed in the long term. In this study, both benefits and complications should be interpreted with caution, as they are based on a small subgroup of treated patients (*n* = 24) and are therefore exploratory rather than conclusive, since data heterogeneity does not allow for comparative or inferential analysis. Additionally, several covariates were not explored, including the duration of the orthodontic treatment. Detailed information regarding this group is presented in the Supplementary Table S2.

Recommendations regarding orthodontic appliance selection in people living with EB can be found in the current CPG [24]. This CPG developed through a Delphi consensus of experts stated that there is no preferable appliance for a specific EB type, as most appliances present specific benefits and complications, with limitations varying according to the EB phenotype severity. Clinical experience regarding aligners in EB patients has also been previously reported [21] and, although aligners may offer benefits regarding ulcer prevention, they still present several limitations, including the cost, difficulties bonding the attachments in the posterior areas and the impossibility to obtain clear scans or even dental impressions in patients with severe microstomia. Other appliances, such as lingual braces, have scarce to no evidence in the EB population. Although lingual appliances might theoretically reduce trauma to the labial and buccal mucosa, this might simply shift the burden, as tongue fragility is equally present in this population. Further studies are still needed to develop specific recommendations considering the EB phenotype and orthodontic needs.

### 4.3. Limitations of This Study

Due to the observational nature of this study, the results must be interpreted with caution, as no causal relationships can be inferred. The findings reflect the self-perceived barriers, complications, and benefits of orthodontic treatment in a specific group of patients with EB who had access to highly specialised dental and orthodontic care. Consequently, the experiences reported in this study may not be fully representative of the broader EB population. In Chile, patients with EB can benefit from the long-standing agreement between DebRA Chile and the Faculty of Dentistry of the University of Chile, which substantially decreases barriers such as financial burden, limited access to specialised care, and lack of trained professionals. This unique healthcare context likely influenced both access to orthodontic treatment and patients’ perceptions of its feasibility and benefits. Therefore, the results may not be directly transferable to healthcare systems in other countries, where access to multidisciplinary and EB-experienced dental care is more limited. Additionally, selection bias could be reflected in those patients who declined to participate and those who could not travel to the dental clinic during the examination period. Further studies in other countries would help enrich the discussion on this topic, as orthodontic treatment in EB is still mainly limited to case reports.

Another important limitation is the reliance on self-reported data. Self-perceived barriers, complications, and benefits may be influenced by recall bias, social desirability bias, or patients’ current treatment outcomes and overall satisfaction. Perceptions may vary depending on the time elapsed since orthodontic treatment, which was not uniform across participants. Although clinical measures were assessed as part of the dental consultation and used to compare with self-perceived oral health status, objective clinical measures for orthodontic benefits and complications were not available, limiting the possibility to validate patient-reported experiences against clinical findings. Selection bias must also be considered, as participants who underwent or considered orthodontic treatment and agreed to participate in the study may represent a subgroup of patients who are more engaged with healthcare services or who had more positive experiences. Patients with less severe disease manifestations, negative treatment experiences, or limited access to care may be underrepresented.

Furthermore, the heterogeneity of EB subtypes and disease severity constitutes an additional limitation. Differences in oral involvement, mucosal fragility, and functional limitations across EB subtypes may significantly influence orthodontic experiences. However, the sample size did not allow for robust subgroup analyses, limiting the ability to explore these differences in detail.

Finally, orthodontic treatment in patients with EB remains a relatively underexplored field, with the existing literature largely restricted to case reports and small case series. While this study contributes novel patient-reported insights, further research—including multicentre studies, international comparisons, and the integration of clinical outcome measures—is necessary to strengthen the evidence base and improve the generalizability of findings.

## 5. Conclusions and Recommendations

Patients perceive the benefits of orthodontic treatment to include long-term improvement in aesthetics, oral hygiene and occlusal stability. Short-term complications include traumatic ulcers, debonding of the appliances/brackets and gingivitis. Long-term complications include caries and enamel demineralisation. As an observational study, barriers are affected by the environment. Thus, further studies in other countries may enrich the analysis and improve access to timely orthodontic treatment for these patients. It is fundamental to take action to decrease these access barriers and incorporate orthodontists as part of the multidisciplinary special care teams. Therefore, dental specialists must be prepared to treat patients with special healthcare needs.

## Figures and Tables

**Table 1:** Self-reported barriers to orthodontic treatment in patients with EB.

EB Type	N	EB-Related and Oral Health Barriers						Psychosocial Barriers				Professional Barriers		Other
		Wounds [*n* (%)]	Pain [*n* (%)]	Limited Mouth Opening [*n* (%)]	Poor Oral Hygiene [*n* (%)]	Poor Oral Health Status [*n* (%)]	Poor Systemic Health Status [*n* (%)]	Patient Behaviour [*n* (%)]	Fear of Wounds or Complications [*n* (%)]	Difficulties Accessing the Clinic [*n* (%)]	Did Not Perceive Benefit [*n* (%)]	Financial Barrier [*n* (%)]	First Orthodontic Evaluation [*n* (%)]	Other Barriers [*n* (%)]
EBS	26	0 (0)	1 (3.8)	0 (0)	3 (11.5)	4 (15.4)	0 (0)	2 (7.7)	1 (3.8)	9 (34.6)	11 (42.3)	1 (3.8)	23 (88.5)	0 (0)
JEB	6	1 (16.7)	1 (16.7)	0 (0)	0 (0)	0 (0)	0 (0)	1 (16.7)	0 (0)	2 (33.3)	1 (16.7)	1 (16.7)	4 (66.7)	0 (0)
DDEB	18	1 (5.6)	1 (5.6)	0 (0)	1 (5.6)	2 (11.1)	0 (0)	1 (5.6)	0 (0)	2 (11.1)	8 (44.4)	0 (0)	12 (66.7)	1 (5.6)
RDEB	49	6 (12.2)	5 (10.2)	18 (36.7)	23 (46.9)	19 (38.8)	2 (4.1)	10 (20.4)	0 (0)	28 (57.1)	6 (12.2)	5 (10.2)	34 (69.4)	5 (10.2)
KEB	2	0 (0)	0 (0)	0 (0)	1 (50.0)	2 (100)	1 (50.0)	1 (50.0)	0 (0)	2 (100)	1 (50.0)	0 (0)	2 (100)	0 (0)
TOTAL	101	8 (7.9)	8 (7.9)	18 (17.8)	28 (27.7)	27 (26.7)	3 (3.0)	15 (14.9)	1 (1.0)	43 (42.6)	27 (26.7)	7 (6.9)	75 (74.3)	6 (5.9)

EBS: Epidermolysis Bullosa Simplex; JEB: Junctional Epidermolysis Bullosa, DDEB: Dominant Dystrophic Epidermolysis Bullosa, RDEB: Recessive Dystrophic Epidermolysis Bullosa, KEB: Kindler Epidermolysis Bullosa.

**Table 2:** Significant associations between EB types and barriers.

	Limited Mouth Opening		Poor Oral Hygiene		Poor Oral Health Status		Difficulties Accessing the Clinic		Does Not Perceive Benefit	
	No	Yes	No	Yes	No	Yes	No	Yes	No	Yes
EBS	100.00	0.00	88.46	11.54	84.62	15.38	65.38	34.62	57.69	42.30
JEB	100.00	0.00	100.00	0.00	100.00	0.00	66.67	33.33	83.33	16.67
DDEB	100.00	0.00	94.44	5.56	88.89	11.11	88.89	11.11	55.55	44.44
RDEB	63.36	36.73	53.06	46.94	61.22	38.78	40.82	59.18	87.75	12.25
KEB	100.00	0.00	50.00	50.00	0.00	100.00	50.00	50.00	50.00	50.00
*Adjusted p value* *(Cramér’s V)*	0.0017 (0.44)		0.0048 (0.39)		0.0404 (0.33)		0.0371 (0.31)		0.0436 (0.29)	

EB: Epidermolysis Bullosa; EBS: EB Simplex; JEB: Junctional EB; DDEB: Dominant Dystrophic EB; RDEB; Recessive Dystrophic EB; KEB: Kindler EB; reported frequencies represent row percentages (conditional proportions by row). Adjusted *p*-values are provided. Cramér’s V is calculated as a measure of effect size.

**Table 3:** Complications and benefits of orthodontic treatment in patients with EB.

Complications and Benefits		*n* (%)
Patients that received orthodontic treatment		24 (100)
**EB type and subtype**	EBS—Localised	1 (4.1)
	EBS—Intermediate	1 (4.1)
	JEB—Intermediate	1 (4.1)
	JEB—Severe	1 (4.1)
	DDEB—Localised	6 (25.0)
	RDEB—Intermediate	6 (25.0)
	RDEB—Severe	7 (29.1)
	RDEB—Inversa	1 (4.1)
**Benefits**		
Aesthetics		15 (62.5)
Oral hygiene		5 (20.8)
Occlusal stability		3 (12.5)
Fewer ulcers		2 (8.3)
Traction allowed oral rehabilitation		1 (4.1)
Improved oral function		1 (4.1)
More space for tongue		1 (4.1)
Complications		
Showed any complication		20 (83.3)
No complications		4 (16.6)
**Oral health**	More wounds or lesions	18 (75.0)
	Gingivitis	13 (54.1)
	Poor oral hygiene	10 (41.6)
	Caries	8 (33.3)
**Orthodontics**	Debonding appliances and brackets	7 (29.1)
	Archwire complications	5 (20.8)
	Suspended treatment	5 (20.8)
	Patient does not follow instructions	4 (16.6)
**Other**	Poor compliance with appointments due to other health issues	1 (4.1)

EBS: Epidermolysis Bullosa Simplex; JEB: Junctional Epidermolysis Bullosa, DDEB: Dominant Dystrophic Epidermolysis Bullosa, RDEB: Recessive Dystrophic Epidermolysis Bullosa.

## Data Availability

The data is only available upon reasonable request. The data are not publicly available due to privacy restriction.

## Supplementary Materials

The following supporting information can be downloaded at: https://www.mdpi.com/article/10.3390/healthcare14111584/s1, Supplementary File S1: Semi-structured interview (Translated from Spanish); Supplementary File S2: STROBE Statement for cross-sectional studies; Supplementary Table S1: Self-Reported barriers associated with orthodontic treatment in patients with EB. Extended version with EB subtypes; Supplementary Table S2: Benefits and complications of orthodontic treatment.

## References

[1] Has C., Bauer J.W., Bodemer C., Bolling M.C., Bruckner-Tuderman L., Diem A., Fine J.-D., Heagerty A., Hovnanian A., Marinkovich M.P. (2020). Consensus reclassification of inherited epidermolysis bullosa and other disorders with skin fragility. Br. J. Dermatol..

[2] Bardhan A., Bruckner-Tuderman L., Chapple I.L.C., Fine J.-D., Harper N., Has C., Magin T.M., Marinkovich M.P., Marshall J.F., McGrath J.A. (2020). Epidermolysis bullosa. Nat. Rev. Dis. Prim..

[3] Abboud L., Leclerc-Mercier S., Bodemer C., Guéro S. (2022). Hand surgery in recessive dystrophic epidermolysis bullosa: Our experience with dermal substitutes. J. Plast. Reconstr. Aesthetic Surg..

[4] Goldschneider K.R., Good J., Harrop E., Liossi C., Lynch-Jordan A., Martinez A.E., Maxwell L.G., Stanko-Lopp D. (2014). Pain care for patients with epidermolysis bullosa: Best care practice guidelines. BMC Med..

[5] Krämer S., Lucas J., Gamboa F., Peñarrocha Diago M., Peñarrocha Oltra D., Guzmán-Letelier M., Paul S., Molina G., Sepúlveda L., Araya I. (2020). Clinical practice guidelines: Oral health care for children and adults living with epidermolysis bullosa. Spéc. Care Dent..

[6] Krämer S., Fuentes I., Yubero M.J., Encina C., Farfán J., Araya I., Bennett J.C., Fuentes C., McNab M.E., Zillmann G. (2020). Absence of tongue papillae as a clinical criterion for the diagnosis of generalized recessive dystrophic epidermolysis bullosa types. J. Am. Acad. Dermatol..

[7] Serrano-Martínez M., Bagán J., Silvestre F., Viguer M. (2003). Oral lesions in recessive dystrophic epidermolysis bullosa. Oral Dis..

[8] Stellingsma C., Dijkstra P.U., Dijkstra J., Duipmans J.C., Jonkman M.F., Dekker R. (2011). Restrictions in oral functions caused by oral manifestations of epidermolysis bullosa. Eur. J. Dermatol..

[9] Wright J., Johnson L., Fine J.-D. (1993). Developmental defects of enamel in humans with hereditary epidermolysis bullosa. Arch. Oral Biol..

[10] Urzúa B., Krämer S., Morales-Bozo I., Camacho C., Yubero M.J., Palisson F., Fuentes I., Ortega-Pinto A. (2021). Case Report: Crown Resorption in a Patient with Junctional Epidermolysis Bullosa and Amelogenesis Imperfecta with LAMB3 Gene Mutations. Front. Dent. Med..

[11] Besa-Witto C., Ortega-Pinto A., Véliz S., Cornejo M., Fuentes I., Palisson F., Krämer S. (2025). Prevalence of Crown Resorption in Amelogenesis Imperfecta due to Junctional Epidermolysis Bullosa. Oral Dis..

[12] Krämer S., Hillebrecht A.L., Wang Y., Badea M.-A., Barrios J.I., Danescu S., Fuentes I., Kartal D., Klausegger A., de León E.P. (2024). Orofacial Anomalies in Kindler Epidermolysis Bullosa. JAMA Dermatol..

[13] Wiebe C.B., Silver J.G., Larjava H.S. (1996). Early-Onset Periodontitis Associated with Weary-Kindler Syndrome: A Case Report. J. Periodontol..

[14] Wiebe C.B., Penagos H., Luong N., Slots J., Epstein E., Siegel D., Häkkinen L., Putnins E.E., Larjava H.S. (2003). Clinical and Microbiologic Study of Periodontitis Associated with Kindler Syndrome. J. Periodontol..

[15] Shah H., McDonald F., Lucas V., Ashley P., Roberts G. (2002). A cephalometric analysis of patients with recessive dystrophic epidermolysis bullosa. Angle Orthod..

[16] Goldschmied F. (1999). Orthodontic management of a patient with Epidermolysis Bullosa. Australas. Orthod. J..

[17] Pacheco W., Marques De Sousa Araugio R. (2008). Orthodontic treatment of a patient with recessive dystrophic epidermolysis bullosa: A case report. Spéc. Care Dent..

[18] Nava E.P., de la Teja Ángeles E., Gutiérrez A.D. (2014). Manejo estomatológico de la maloclusión dental en los pacientes con epidermólisis bullosa distrófica mediante la guía interceptiva de la oclusión (GIO): Comparación de dos casos. Rev. Mex. Ortodon..

[19] Blanchet I., Tardieu C., Casazza E. (2021). Oral Care in Kindler Syndrome: 7-Year Follow-up of 2 Brothers. J. Clin. Pediatr. Dent..

[20] Véliz S., Huber H., Yubero M.J., Fuentes I., Alsayer F., Krämer S.M. (2020). Early teeth extraction in patients with generalized recessive dystrophic epidermolysis bullosa: A case series. Spéc. Care Dent..

[21] Véliz Méndez S., Baeza M., Krämer Strenger S. (2023). Impression technique modification and oral contracture release surgery for orthodontic treatment in a patient with severe microstomia due to recessive dystrophic epidermolysis bullosa. Spéc. Care Dent..

[22] Véliz Méndez S., Baeza Paredes M., Olivares A., Vicuña M.J., Krämer Strenger S.M. (2024). Comprehensive orthodontic treatment using miniscrews and digital rehabilitation in a patient with severe recessive dystrophic epidermolysis bullosa. Spéc. Care Dent..

[23] Véliz S., Olivares A., Krämer S. (2024). Mini-implant assisted palate expansion and digital design in junctional epidermolysis bullosa and amelogenesis imperfecta: Case report. Spéc. Care Dent..

[24] Véliz S., Abeleira M.T., Serrano M.C., Charavet C., FitzGerald K., Hormazábal F., Huber H., Korolenkova M., Mubeen S., Paul S. (2025). Orthodontic Treatment in Patients with Epidermolysis Bullosa (EB)—Clinical Practice Guidelines (CPG). Spéc. Care Dent..

[25] Méndez S.V., Rotman M., Hormazábal F., Sepúlveda L., Valle M., Álvarez E. (2022). Barriers and facilitators in the orthodontic treatment of teenagers with neurodevelopmental disabilities. Am. J. Orthod. Dentofac. Orthop..

[26] AlSarheed M., Bedi R., Alkhatib M.N., Hunt N.P. (2006). Dentists’ Attitudes and Practices Toward Provision of Orthodontic Treatment for Children with Visual and Hearing Impairments. Spéc. Care Dent..

[27] Arriagada-Vargas C., Abeleira-Pazos M.T., Outumuro-Rial M., García-Mato E., Varela-Aneiros I., Limeres-Posse J., Diz-Dios P., Diniz-Freitas M. (2022). Rare Disorders: Diagnosis and Therapeutic Planning for Patients Seeking Orthodontic Treatment. J. Clin. Med..

[28] Becker A., Shapira J., Chaushu S. (2009). Orthodontic treatment for the special needs child. Prog. Orthod..

[29] Von Elm E., Altman D.G., Egger M., Pocock S.J., Gøtzsche P.C., Vandenbroucke J.P., Initiative S. (2007). The Strengthening the Reporting of Observational Studies in Epidemiology (STROBE) statement: Guidelines for reporting observational studies. Lancet.

[30] Benjamini Y., Hochberg Y. (1995). Controlling the False Discovery Rate: A Practical and Powerful Approach to Multiple Testing. J. R. Stat. Soc. Ser. B Methodol..

[31] Palisson F., Yubero M.J., Lecaros C., Krämer S., Fuentes C., Morandé P., Noya B., Cofré G., Castillo J., Acevedo F. (2024). Epidemiology of epidermolysis bullosa in Chile. Br. J. Dermatol..

[32] World Health Organization (2007). International Classification of Functioning Disability and Health (ICF).

[33] Sanders N.L. (1999). Evidence-Based Care in Orthodontics and Periodontics: A Review of the Literature. J. Am. Dent. Assoc..

[34] Wiener R.C., Dwibedi N., Shen C., Findley P.A., Sambamoorthi U. (2018). Clinical Oral Health Recommended Care and Oral Health Self-Report, NHANES, 2013–2014. Adv. Public Health.

[35] Johal A., Damanhuri S.H., Colonio-Salazar F. (2024). Adult orthodontics, motivations for treatment, choice, and impact of appliances: A qualitative study. Am. J. Orthod. Dentofac. Orthop..

[36] MacIsaac M.F., Wright J.M., Le N.K., Pringle A.J., Schuster L.A., Brown A.B., Kochenour W.L., Crisp T.O., Halsey J.N., Rottgers S.A. (2025). Barriers in Accessing Orthodontic Care for Patients with Orofacial Clefts: Insights from a Florida-Based Survey and National Database Analysis. Cleft Palate Craniofacial J..

[37] Brown B.R., Inglehart M.R. (2009). Orthodontists’ and orthodontic residents’ education in treating underserved patients: Effects on professional attitudes and behavior. J. Dent. Educ..

[38] Krämer S., Hillebrecht A.L., Bekes K., Bücher K., Clark V., Haririan H., Jakowski J., Joseph C., Meißner N., Monteiro J. (2025). Oral health care pathways for patients with epidermolysis bullosa: A position statement from the European reference network for rare skin diseases. J. Eur. Acad. Dermatol. Venereol..

[39] Becker A., Shapira J., Chaushu S. (2000). Orthodontic treatment for disabled children: Motivation, expectation, and satisfaction. Eur. J. Orthod..

[40] Abeleira M.T., Pazos E., Limeres J., Outumuro M., Diniz M., Diz P. (2016). Fixed multibracket dental therapy has challenges but can be successfully performed in young persons with Down syndrome. Disabil. Rehabil..

[41] Miranda Galvis M., Faustino I.S.P., Ferraz F.C., Castelli Sanchez F.J., Santos-Silva A.R., Lopes M.A. (2020). Orthodontic treatment in a patient with cherubism: Benefits and limitations. Spéc. Care Dent..

[42] AlDahash F., AlShamali D., AlBander W., Bakhsh R., AlMadhi W., AlSenani S. (2020). Oral mucosal ulceration during orthodontic treatment: The perception of patients and knowledge and attitude of the orthodontic practitioners. J. Fam. Med. Prim. Care.

[43] Chang J., Li X. (2024). Multivariate analysis of oral mucosal ulcers during orthodontic treatment. World J. Clin. Cases.

[44] Jepsen K., Sculean A., Jepsen S. (2023). Complications and treatment errors involving periodontal tissues related to orthodontic therapy. Periodontol. 2000.

