# Supplementary material for: Barriers, Benefits and Complications of Orthodontic Treatment in Patients with Epidermolysis Bullosa: A Patient-Reported Cross-Sectional Study
Source: Healthcare (Basel). 2026 Jun 4;14(11):1584. doi: 10.3390/healthcare14111584 (PMC13256972; doi:10.3390/healthcare14111584)
Supplement: Supplementary file 1 [file healthcare-14-01584-s001.zip › healthcare-4319414-supplementary.pdf]

## SUPPLEMENTARY MATERIALS

**Supplementary File S1:** Semi-structured interview (Translated from Spanish).

1. Have you used any type of orthodontic treatment?
   If yes,  What barrier(s) have you faced or experienced?
   If no, (somebody answer "I have never used orthodontic treatment"): Why?

   EB-related and oral health barriers:
   - Wounds
   - Pain
   - Limited mouth opening
   - Poor oral hygiene
   - Poor oral health status
   - Poor systemic health status

   Psychosocial barriers:
   - Patient behaviour
   - Fear of wounds/complications
   - Difficulties accessing the clinic
   - Does not consider it necessary or beneficial

   Professional barriers
   - Financial barrier
   - 1st orthodontic evaluation
   - Poor treatment prognosis

   Other
   - Another barrier: Write the barrier.
2. For those who have used orthodontic treatment: "Did you experience any complication during your orthodontic treatment?"
   If yes, "What complications did you experienced during your orthodontic treatment?

   Oral/systemic complication:
   - Wounds or lesions
   - Gingivitis
   - Poor oral hygiene
   - Caries

   Orthodontic complications:
   - Debonding appliances or brackets
   - Arch wire complications
   - Treatment suspension
   - Patient does not follow instructions

   Other complication: Write the complication.
3. For those who have used orthodontic treatment: "What benefit did you experience after receiving orthodontic treatment?"

   Benefits:
   - Oral hygiene
   - Aesthetics
   - Oral function improvement
   - Less oral blisters
   - Other benefit: Write the benefit.

**Supplementary File S2:** STROBE Statement for cross-sectional studies

| | Item No | Recommendation | Page |
|---|---|---|---|
| **Title and abstract** | 1 | (*a*) Indicate the study's design with a commonly used term in the title or the abstract | Page 1, line 4. |
| | | (*b*) Provide in the abstract an informative and balanced summary of what was done and what was found | Page 1, line 25 |
| **Introduction** | | | |
| Background/rationale | 2 | Explain the scientific background and rationale for the investigation being reported | Page 3, line 2 |
| Objectives | 3 | State specific objectives, including any prespecified hypotheses | Page 3, line 46 |
| **Methods** | | | |
| Study design | 4 | Present key elements of study design early in the paper | Page 4, line 4 |
| Setting | 5 | Describe the setting, locations, and relevant dates, including periods of recruitment, exposure, follow-up, and data collection | Page 4, line 4 |
| Participants | 6 | (*a*) Give the eligibility criteria, and the sources and methods of selection of participants | Page 4, line 5 |
| Variables | 7 | Clearly define all outcomes, exposures, predictors, potential confounders, and effect modifiers. Give diagnostic criteria, if applicable | Page 4, line 17 |
| Data sources/ measurement | 8* | For each variable of interest, give sources of data and details of methods of assessment (measurement). Describe comparability of assessment methods if there is more than one group | Page 4, line 17 |
| Bias | 9 | Describe any efforts to address potential sources of bias | Page 4, line 36 |
| Study size | 10 | Explain how the study size was arrived at | Page 4, line 43 |
| Quantitative variables | 11 | Explain how quantitative variables were handled in the analyses. If applicable, describe which groupings were chosen and why | Page 4, line 48 |
| Statistical methods | 12 | (*a*) Describe all statistical methods, including those used to control for confounding | Page 4, line 48 |
| | | (*b*) Describe any methods used to examine subgroups and interactions | Page 4, line 48 |
| | | (*c*) Explain how missing data were addressed | NA |
| | | (*d*) If applicable, describe analytical methods taking account of sampling strategy | Page 4, line 48 |
| | | (*e*) Describe any sensitivity analyses | NA |
| **Results** | | | |
| Participants | 13* | (a) Report numbers of individuals at each stage of study—eg numbers potentially eligible, examined for eligibility, confirmed eligible, included in the study, completing follow-up, and analysed | Page 5, line 16 |
| | | (b) Give reasons for non-participation at each stage | Figure 1 |
| | | (c) Consider use of a flow diagram | Figure 1 |
| Descriptive data | 14* | (a) Give characteristics of study participants (eg demographic, clinical, social) and information on exposures and potential confounders | Page 5, line 16 |
| | | (b) Indicate number of participants with missing data for each variable of interest | NA |
| Outcome data | 15* | Report numbers of outcome events or summary measures | Table 1 |

| | | | |
|---|---|---|---|
| Main results | 16 | (*a*) Give unadjusted estimates and, if applicable, confounder-adjusted estimates and their precision (eg, 95% confidence interval). Make clear which confounders were adjusted for and why they were included | Page 6, line 6 |
| | | (*b*) Report category boundaries when continuous variables were categorized | Page 6, line 6 |
| | | (*c*) If relevant, consider translating estimates of relative risk into absolute risk for a meaningful time period | NA |
| Other analyses | 17 | Report other analyses done—eg analyses of subgroups and interactions, and sensitivity analyses | Figure 2 |
| **Discussion** | | | |
| Key results | 18 | Summarise key results with reference to study objectives | Page 11, line 3 |
| Limitations | 19 | Discuss limitations of the study, taking into account sources of potential bias or imprecision. Discuss both direction and magnitude of any potential bias | Page 14, line 28 |
| Interpretation | 20 | Give a cautious overall interpretation of results considering objectives, limitations, multiplicity of analyses, results from similar studies, and other relevant evidence | Page 11, line 6 |
| Generalisability | 21 | Discuss the generalisability (external validity) of the study results | Page 11, line 6 |
| **Other information** | | | |
| Funding | 22 | Give the source of funding and the role of the funders for the present study and, if applicable, for the original study on which the present article is based | Page 4, line 13 |

*Give information separately for exposed and unexposed groups.

**Note:** An Explanation and Elaboration article discusses each checklist item and gives methodological background and published examples of transparent reporting. The STROBE checklist is best used in conjunction with this article (freely available on the Web sites of PLoS Medicine at http://www.plosmedicine.org/, Annals of Internal Medicine at http://www.annals.org/, and Epidemiology at http://www.epidem.com/). Information on the STROBE Initiative is available at www.strobe-statement.org.

Supplementary Table S1: Self-Reported barriers associated with orthodontic treatment in patients with EB. Extended version with EB subtypes.

| EB TYPE | EB SUBTYPE | N | EB-RELATED AND ORAL HEALTH BARRIERS | | | | | | PSYCHOSOCIAL BARRIERS | | | | PROFESSIONAL BARRIERS | | OTHER |
|---|---|---|---|---|---|---|---|---|---|---|---|---|---|---|---|
| | | | Wounds [n (%)] | Pain [n (%)] | Limited mouth opening [n (%)] | Poor oral hygiene [n (%)] | Poor oral health status [n (%)] | Poor systemic health status [n (%)] | Patient behaviour [n (%)] | Fear of wounds or complications [n (%)] | Difficulties to access the clinic [n (%)] | Did not consider it necessary or did not see benefit [n (%)] | Financial barrier [n (%)] | First orthodontic evaluation [n (%)] | Other barriers [n (%)] |
| EBS | Localised | 3 | 0(0) | 0(0) | 0(0) | 0(0) | 0(0) | 0(0) | 0(0) | 0(0) | 1(33.3) | 2(66.7) | 0(0) | 2(66.7) | 0(0) |
| | Intermediate | 18 | 0(0) | 1(5.6) | 0(0) | 0(0) | 2(11.1) | 0(0) | 1(5.6) | 1(5.6) | 3(16.7) | 9(50.0) | 0(0) | 16(88.9) | 0(0) |
| | IWMD | 5 | 0(0) | 0(0) | 0(0) | 3(60.0) | 2(40.0) | 0(0) | 1(20.0) | 0(0) | 5(100) | 0(0) | 1(20.0) | 5(100) | 0(0) |
| | *EBS* | 26 | 0(0) | 1(3.8) | 0(0) | 3(11.5) | 4(15.4) | 0(0) | 2(7.7) | 1(3.8) | 9(34.6) | 11(42.3) | 1(3.8) | 23 (88.5) | 0(0) |
| JEB | Intermediate | 3 | 0(0) | 0(0) | 0(0) | 0(0) | 0(0) | 0(0) | 1(33.3) | 0(0) | 0(0) | 1(33.3) | 1(33.3) | 2(66.7) | 0(0) |
| | Severe | 3 | 1(33.3) | 1(33.3) | 0(0) | 0(0) | 0(0) | 0(0) | 0(0) | 0(0) | 2(66.7) | 0(0) | 0(0) | 2(66.7) | 0(0) |
| | *JEB* | 6 | 1(16.7) | 1(16.7) | 0(0) | 0(0) | 0(0) | 0(0) | 1(16.7) | 0(0) | 2(33.3) | 1(16.7) | 1(16.7) | 4(66.7) | 0(0) |
| DDEB | Localised | 14 | 1(7.1) | 1(7.1) | 0(0) | 1(7.1) | 2(14.3) | 0(0) | 1(7.1) | 0(0) | 2(14.3) | 5(35.7) | 0(0) | 8(57.1) | 1(7.1) |
| | Pruriginous | 4 | 0(0) | 0(0) | 0(0) | 0(0) | 0(0) | 0(0) | 0(0) | 0(0) | 0(0) | 3(75.0) | 0(0) | 4(100) | 0(0) |
| | *DDEB* | 18 | 1(5.6) | 1(5.6) | 0(0) | 1(5.6) | 2(11.1) | 0(0) | 1(5.6) | 0(0) | 2(11.1) | 8(44.4) | 0(0) | 12(66.7) | 1(5.6) |
| RDEB | Localised | 1 | 0(0) | 0(0) | 0(0) | 0(0) | 1(100) | 0(0) | 0(0) | 0(0) | 0(0) | 1(100) | 0(0) | 1(100) | 0(0) |
| | Intermediate | 21 | 2(9.5) | 3(14.3) | 3(14.3) | 4(19.0) | 3(14.3) | 0(0) | 8(38.1) | 0(0) | 7(33.3) | 4(19.0) | 2(9.5) | 15(71.4) | 1(4.8) |
| | Severe | 25 | 3(12.0) | 1(3.7) | 14(56.0) | 19(76.0) | 14(56.0) | 2(8.0) | 2(8.0) | 0(0) | 20(80.0) | 1(4.0) | 3(12.0) | 17(68.0) | 4(16.0) |
| | Inversa | 2 | 1(50.0) | 1(50.0) | 1(50.0) | 0(0) | 1(50.0) | 0(0) | 0(0) | 0(0) | 1(50.0) | 0(0) | 0(0) | 1(50.0) | 0(0) |
| | *RDEB* | 49 | 6(12.2) | 5(10.2) | 18(36.7) | 23(46.9) | 19(38.8) | 2(4.1) | 10(20.4) | 0(0) | 28(57.1) | 6(12.2) | 5(10.2) | 34(69.4) | 5(10.2) |
| KEB | Kindler | 2 | 0(0) | 0(0) | 0(0) | 1(50.0) | 2(100) | 1(50.0) | 1(50.0) | 0(0) | 2(100) | 1(50.0) | 0(0) | 2(100) | 0(0) |
| TOTAL | | 101 | 8(7.9) | 8(7.9) | 18(17.8) | 28(27.7) | 27(26.7) | 3(3.0) | 15(14.9) | 1(1.0) | 43(42.6) | 27(26.7) | 7(6.9) | 75(74.3) | 6(5.9) |

EBS: Epidermolysis Bullosa Simplex; JEB: Junctional Epidermolysis Bullosa, DDEB: Dominant Dystrophic Epidermolysis Bullosa, RDEB: Recessive Dystrophic Epidermolysis Bullosa. IWMD: EB Simplex Intermediate with Muscular Dystrophy.

Supplementary Table S2: Benefits and complications of orthodontic treatment.

| | | | | Benefits | | | | | | | Complications | | | | | | | | |
|---|---|---|---|---|---|---|---|---|---|---|---|---|---|---|---|---|---|---|---|
| | | | | | | | | | | | Oral health | | | | Orthodontics | | | | Other |
| EB type | EB subtype | Type of treatment | Appliance | Aesthetic improvement | Better oral hygiene | Occlusal stability | Fewer ulcers | Traction allowed oral rehabilitation | Improved oral function | More space for tongue | More wounds or lesions | Gingivitis | Poor oral hygiene | Caries | Debonding appliances and brackets | Archwire complication | Suspended treatment | Patient does not follow instructions | Poor compliance with appointments due to other health issues |
| EBS | Localised | Orthopaedic | Removable (Trainer) | 1 | 0 | 0 | 0 | 0 | 0 | 0 | 0 | 0 | 0 | 0 | NA | NA | 0 | 0 | 0 |
| EBS | Intermediate | Orthodontic | Fixed (Metal braces) | 1 | 1 | 0 | 0 | 0 | 0 | 0 | 1 | 0 | 0 | 0 | 0 | 0 | 0 | 0 | 0 |
| JEB | Intermediate | Combined | Fixed (Palatal expander + metal braces + mini implant) | 1 | 0 | 0 | 0 | 0 | 1 | 0 | 1 | 1 | 1 | 0 | 1 | 0 | 0 | 0 | 0 |
| JEB | Severe | Orthodontic | Fixed (Metal braces) | 0 | 0 | 0 | 0 | 1 | 0 | 0 | 1 | 1 | 1 | 0 | 1 | 0 | 0 | 0 | 0 |
| DDEB | Localised | Combined | Fixed (Palatal expander + metal braces) | 0 | 0 | 0 | 0 | 0 | 0 | 0 | 0 | 1 | 0 | 0 | 0 | 0 | 0 | 1 | 0 |
| DDEB | Localised | Combined | Removable (Palatal expander) / Fixed (Metal braces) | 1 | 0 | 0 | 0 | 0 | 0 | 0 | 1 | 0 | 0 | 0 | 0 | 0 | 0 | 1 | 0 |
| DDEB | Localised | Orthodontic | Fixed (Metal braces) | 1 | 0 | 0 | 0 | 0 | 0 | 0 | 1 | 1 | 1 | 0 | 1 | 0 | 0 | 0 | 0 |
| DDEB | Localised | Orthopaedic | Fixed (Palatal expander) | 1 | 1 | 1 | 1 | 0 | 0 | 0 | 1 | 1 | 0 | 0 | 1 | 0 | 0 | 0 | 0 |
| DDEB | Localised | Orthodontic | Fixed (Metal braces) | 0 | 1 | 0 | 0 | 0 | 0 | 0 | 1 | 0 | 0 | 0 | 0 | 0 | 0 | 0 | 0 |
| DDEB | Localised | Orthopaedic | Removable (Andresen activator) | 0 | 0 | 0 | 0 | 0 | 0 | 0 | 0 | 0 | 0 | 0 | NA | 0 | 0 | 0 | 0 |
| RDEB | Intermediate | Orthodontic | Fixed (Metal braces) | 1 | 0 | 1 | 0 | 0 | 0 | 0 | 1 | 1 | 1 | 1 | 1 | 0 | 0 | 0 | 0 |
| RDEB | Intermediate | Orthodontic | Fixed (Metal braces) | 1 | 0 | 0 | 0 | 0 | 0 | 0 | 1 | 0 | 0 | 0 | 0 | 0 | 0 | 0 | 0 |
| RDEB | Intermediate | Orthodontic | Fixed (Metal braces) | 1 | 1 | 0 | 0 | 0 | 0 | 0 | 1 | 1 | 0 | 1 | 0 | 1 | 0 | 0 | 0 |
| RDEB | Intermediate | Orthodontic | Fixed (Metal braces) | 0 | 0 | 0 | 0 | 0 | 0 | 0 | 0 | 0 | 0 | 0 | 0 | 0 | 1 | 0 | 0 |
| RDEB | Intermediate | Orthodontic | Removable (Aligners) | 0 | 0 | 0 | 0 | 0 | 0 | 0 | 0 | 0 | 0 | 0 | NA | NA | 0 | 1 | 0 |
| RDEB | Intermediate | Orthopaedic | Removable (Palatal expander) / Fixed (Palatal expander) | 0 | 0 | 0 | 0 | 0 | 0 | 0 | 1 | 0 | 0 | 0 | 0 | 0 | 1 | 1 | 0 |
| RDEB | Severe | Orthodontic | Fixed (Metal braces) | 1 | 0 | 0 | 1 | 0 | 0 | 0 | 1 | 1 | 1 | 1 | 0 | 1 | 1 | 0 | 0 |

Supplementary Table 2: Continuation.

| EB type | EB subtype | Type of treatment | Appliance | Benefits | | | | | | | Complications | | | | | | | | |
|---|---|---|---|---|---|---|---|---|---|---|---|---|---|---|---|---|---|---|---|
| | | | | | | | | | | | Oral health | | | | Orthodontics | | | | Other |
| | | | | Aesthetic improvement | Better oral hygiene | Occlusal stability | Fewer ulcers | Traction allowed oral rehabilitation | Improved oral function | More space for tongue | More wounds or lesions | Gingivitis | Poor oral hygiene | Caries | Debonding appliances and brackets | Archwire complication | Suspended treatment | Patient does not follow instructions | Poor compliance with appointments due to other health issues |
| RDEB | Severe | Orthodontic | Fixed (Metal braces + mini implant) | 1 | 1 | 1 | 0 | 0 | 0 | 0 | 1 | 1 | 1 | 1 | 0 | 1 | 0 | 0 | 0 |
| RDEB | Severe | Orthodontic | Fixed (Metal braces + mini implant) | 1 | 0 | 0 | 0 | 0 | 0 | 0 | 1 | 1 | 1 | 1 | 0 | 1 | 0 | 0 | 0 |
| RDEB | Severe | Orthodontic | Removable (Aligners) | 1 | 0 | 0 | 0 | 0 | 0 | 1 | 0 | 0 | 0 | 0 | NA | NA | 0 | 0 | 0 |
| RDEB | Severe | Orthodontic | Fixed (Metal braces) | 1 | 0 | 0 | 0 | 0 | 0 | 0 | 1 | 1 | 1 | 1 | 0 | 0 | 1 | 0 | 0 |
| RDEB | Severe | Orthodontic | Fixed (Metal braces) | 0 | 0 | 0 | 0 | 0 | 0 | 0 | 1 | 1 | 1 | 1 | 1 | 1 | 1 | 0 | 1 |
| RDEB | Severe | Orthodontic | Fixed (Metal braces) | 1 | 0 | 0 | 0 | 0 | 0 | 0 | 1 | 1 | 1 | 1 | 1 | 0 | 0 | 0 | 0 |
| RDEB | Inversa | Orthodontic | Fixed (Metal braces) | 0 | 0 | 0 | 0 | 0 | 0 | 0 | 1 | 0 | 0 | 0 | 0 | 0 | 0 | 0 | 0 |
| | | | TOTAL (n) | 15 | 5 | 3 | 2 | 1 | 1 | 1 | 18 | 13 | 10 | 8 | 7 | 5 | 5 | 4 | 1 |

EBS: Epidermolysis Bullosa Simplex; JEB: Junctional Epidermolysis Bullosa, DDEB: Dominant Dystrophic Epidermolysis Bullosa, RDEB: Recessive Dystrophic Epidermolysis Bullosa. 0=no, 1=yes, NA: Does not apply
